# Ketamine, Transcranial Magnetic Stimulation, and Depression Specific Yoga and Mindfulness Based Cognitive Therapy in Management of Treatment Resistant Depression: Review and Some Data on Efficacy

**DOI:** 10.1155/2015/842817

**Published:** 2015-10-05

**Authors:** Basant Pradhan, Tapan Parikh, Ramkrishna Makani, Madhusmita Sahoo

**Affiliations:** ^1^Cooper University Health System and Cooper Medical School of Rowan University, Camden, NJ, USA; ^2^Department of Psychiatry, Cooper University Health System, 401 Haddon Avenue, E&R Building, Camden, NJ 08103, USA

## Abstract

Depression affects about 121 million people worldwide and prevalence of major depressive disorder (MDD) in US adults is 6.4%. Treatment resistant depression (TRD) accounts for approximately 12–20% of all depression patients and costs $29–$48 billion annually. Ketamine and repetitive transcranial magnetic stimulation (rTMS) have useful roles in TRD, but their utility in long term is unknown. As per the latest literature, the interventions using Yoga and meditation including the mindfulness based cognitive therapy (MBCT) have been useful in treatment of depression and relapse prevention. We present a review of rTMS, ketamine, and MBCT and also report efficacy of a depression specific, innovative, and translational model of Yoga and mindfulness based cognitive therapy (*DepS Y-MBCT*), developed by the first author. *DepS Y-MBCT* as an adjunctive treatment successfully ameliorated TRD symptoms in 27/32 patients in an open label pilot trial in TRD patients. Considering the limitations of existing treatment options, including those of ketamine and rTMS when used as the sole modality of treatment, we suggest a “tiered approach for TRD” by combining ketamine and rTMS (alone or along with antidepressants) for rapid remission of acute depression symptoms and to use *DepS Y-MBCT* for maintaining remission and preventing relapse.

## 1. Introduction

Data from the National Institute of Mental Health indicate that the prevalence of major depressive disorder (MDD) is 6.4% of the US adult population [1] and that a large number of these patients do not respond to currently available treatments including the selective serotonin reuptake inhibitors and the first line antidepressants and eventually result in treatment resistant depression (TRD) [2]. TRD accounts for approximately 12–20% of those who are diagnosed with depression and sums to annual $29–$48 billion in additional costs [3]. Usually there is a delay of weeks to months in treatment response with conventional antidepressants even in cases of successful treatments [4] which is a major drawback and necessitates the development of faster acting antidepressants. This is particularly of paramount importance for depressed patients who present with suicidality, a major challenge in TRD. Thus there is a clear need to develop innovative, rapidly effective, and longer lasting treatments for patients with TRD. Apart from limitations in terms of treatment, an important challenge in the research involving TRD is the limited knowledge and lack of clear and universally acceptable definitions which pose significant difficulties in operationalization, standardization, and generalization of the various treatment approaches for this important public health burden [5]. Often TRD is confused with some rather ill-defined terminologies like “difficult to treat depression” (this generally refers to depression which because of its underlying psychopathology or psychosocial issues or due to nonadherence to treatment is not amenable to treatment) and “pseudoresistance” (this generally refers to treatment failures due to inadequate dosages or inadequate duration of pharmacotherapy or nonadherence to treatment) [6]. Berlim and Turecki, in their systemic review (2007), have identified six different definitions in which the most general definition revolves around nonresponse to more classes of antidepressants. Usually TRD is defined as “a major depressive episode(s) (typically, unipolar depression) that do not respond effectively after two trials of antidepressant monotherapy in adequate dosage and durations (at least 8 weeks, may be 12 weeks in some cases) and often do not respond satisfactorily to numerous sequential treatment regimens” [7]. The European Medicines Agency defined TRD as “a current episode of depressive disorder which has not benefited from at least two adequate trials of antidepressant compounds of different mechanism of action” [8]. All these definitions are limited by the fact that they were mainly based on the pharmacological approaches without taking into consideration the important psychotherapeutic approaches like cognitive behavioral therapy (CBT), interpersonal therapy (IPT), or mindfulness based cognitive therapy (MBCT), and so forth.

In a recent review, Al-Harbi (2012) has summarized the standard of care, therapeutic trends, and the challenges involved in patients with TRD. It is important to note that most of the approved antidepressant medications primarily target the brain monoamine systems (serotonin, norepinephrine, or, in some cases, dopamine) and, unlike ketamine which acts ultrarapidly (usually within 2 hours of infusion or even quicker than this), none of these are known to target the glutamate system which has been implicated as an important therapeutic target based on the recent research in TRD. In general, the therapeutic options for TRD include two major strategies, that is,* augmentation* of antidepressant medication(s) which is done for* partial responders* and* optimization* of antidepressant medication(s) which is used for* nonresponders*. Before using these strategies, a thorough revision of psychiatric and medical diagnoses is necessary not only to identify misdiagnoses, if any, but also to identify any medical and psychiatric comorbidities that could contribute to the treatment resistance. The augmentation strategies involve adding one (or more) agent(s) which is not an antidepressant but may enhance the effect of the antidepressant. These augmenting agents are lithium, thyroid hormones, buspirone, pindolol, psychostimulants, atypical antipsychotics, sex hormones, anticonvulsants/mood stabilizers, and dopamine agonists. In contrast,* optimization* strategies involve maximization of the dose of the antidepressant for adequate time and assessment of serum levels of prescribed antidepressants if indicated. It may also involve switching to another antidepressant(s) (usually from a different class) or using a combination of antidepressants or may include addition of atypical antipsychotics with antidepressant properties. Adequate dosage and duration (usually 6–8 weeks) and adherence must be allowed for these psychotropic agents before they are deemed ineffective. If these agents fail, other approaches involve use of somatic therapies like ECT (electroconvulsive therapy), VNS (vagal nerve stimulation), and rTMS (repetitive transcranial magnetic stimulation) [9]. Use of DBS (deep brain stimulation) in TRD has remained experimental and is usually reserved as last resort for isolated and utterly resistant cases only. Last but not the least, integrated approaches for TRD involve use of antidepressants together with other modes of treatment which include ketamine, psychotherapy, risk management strategies, complementary and alternative medicine (CAM) therapies (including Yoga and mindfulness approaches), and life style changes such as aerobic exercises, stress management, and vacation [10, 11]. Additionally, use of strategies to manage the side effects of antidepressants and other psychotropic agents remains important in treating patients with TRD. Despite these existing strategies, TRD still remains a huge public health burden which includes the burdensome risk of suicide in this population.

## 2. Methods for Review of Literature on Ketamine, rTMS, and Yoga and Mindfulness Interventions in TRD

Considering the severity of TRD and scarcity in the availability of effective and relatively faster acting pharmacological and psychological treatments, it is quite pertinent to look for other treatment options, as sole modality of treatment or as adjunct. It is encouraging to note that there has been considerable progress in this regard which includes the newer and recent potential treatment modalities such as ketamine, rTMS, and Yoga and mindfulness based interventions. We searched literature on this topic using PubMed and the literature search is as up to date as of August 15th, 2015. We restricted our search to English language. For searching literature on ketamine in TRD, our search terminologies included “ketamine in TRD,” “ketamine in treatment resistant depression,” “ketamine and TRD,” “ketamine and depression,” and “ketamine and suicidality.” Literature for rTMS was performed using the search terms “rTMS and depression,” “rTMS and treatment resistant depression,” and “rTMS and resistant depression.” Mindfulness based interventions particularly in TRD were searched using the terms “mindfulness and TRD,” “Mindfulness Based Cognitive Therapy (MBCT) and depression,” “mindfulness and depression,” and “Yoga and depression.”

During our literature review for the available treatment options for TRD, we noticed that the last decade has witnessed not only emergence of novel interventions like ketamine but also the considerable progress which has been made in examining the utility of the other not-so-new interventions like rTMS, Yoga, and mindfulness based cognitive therapy (MBCT). Ketamine, working primarily as a glutamate receptor (NMDA) antagonist, suppresses excitatory effects of glutamate, promotes synaptogenesis in key brain areas involved in depression, and rapidly improves symptoms of TRD including suicidality as discussed later. However, effects of ketamine are short lasting and when it is used in long term settings the risks versus benefits ratios of ketamine treatment for TRD are yet to be known. As elaborated later, rTMS has been studied in TRD but data on its long term role in treatment of depression are not consistent. Although rTMS has many advantages over ECT in terms of its favorable side effect profile, the comparative robustness of its effects is low. Yoga and mindfulness based interventions are important therapeutic approaches that are reported to be safe and effective not only in depression but also in other psychiatric disorders both in adults and in the youth [12]. In the subsequent portions of this paper, we present a summary of the review of contemporary literature on TRD, mostly focusing on ketamine, rTMS, and mindfulness based cognitive therapy (MBCT). In addition, we present data from our pilot study that examined efficacy of* depression specific Yoga and mindfulness based cognitive therapy* (*DepS Y-MBCT*) that was developed by the first author [13]. Because the standard of care options for TRD, as they stand now, are not enough to address the huge burden, utility of these alternative and important approaches, for example, ketamine, rTMS, and Yoga and mindfulness based approaches like* DepS Y-MBCT*, is worth exploring based on the emerging evidences in support of their clinical utility in TRD. Additionally, we propose some innovative ways that include a “stepped care approach” that could possibly circumvent some of the existing therapeutic challenges in treatment of patients suffering from TRD.

## 3. Ketamine in TRD

In this section we present an overview of the various key aspects of ketamine, that is, neurobiology of the glutamate system in depression and role of ketamine (and its metabolites) in inducing remission of symptoms of TRD quite rapidly and role of ketamine in neuroplasticity and synaptogenesis that could possibly explain these rapid effects. In addition we discuss the limitations of ketamine including its short lasting effects and concerns about its side effects some of which interestingly have been postulated as the predictors of its clinical response. Despite clear data on clinical efficacy of ketamine in TRD, many aspects of the underlying mechanisms that came from the recent research are still unclear and warrant further studies for providing more clarity. The major mechanisms of action of ketamine (and its metabolites) in depression are (1) its antagonism of the NMDA subtype of glutamate receptor, (2) its inhibition of the phosphorylation of the eukaryotic elongation factor 2 (eEF2, also called CaMKIII) thus resulting in increased synaptic protein synthesis, (3) its action via the* mammalian target of rapamycin* (mTOR) dependent synapse formation, (4) its inhibition of the nicotinic acetyl choline receptor (NAR), and (5) its suppression of the glycogen synthase kinase 3 (GSK-3) [14, 15]. Ketamine's effect on GSK-3 provides the rationale to combine ketamine with lithium (which suppresses GSK-3 as well) for their possible synergistic effect on treatment resistant depression. Ketamine is primarily known to be an antagonist of the excitatory glutamate receptors: it directly blocks the NMDA subtype of the glutamate receptor and through this it indirectly affects the AMPA receptors, the other subtype. The neurobiological mechanisms that implicate the glutamate system in TRD and the role of ketamine in it are complex and are dependent on many factors including the dose of ketamine and locations of the glutamate receptors not only in the key brain areas implicated in TRD but also with respect to the synaptic junction, that is, presynaptic versus postsynaptic. Many researchers have further elaborated upon the possible dysfunction of glutamatergic signaling contributing to depression by the involvement of D-serine which is a key NMDA receptor coagonist that plays a critical role in long term potentiation and also NMDA-induced toxicity which has been implicated as one of the neurobiological mechanisms of depression [16]. Glutamate evokes the release of D-serine from astrocytes and neurons, which then acts at the glycine site on the NR1 subunit of NMDA receptors that result in excitotoxicity. Ketamine, by its antiglutamate actions, is postulated to reverse this neurotoxicity, promotes rapid proliferation of the dendritic spines in key brain areas, and thus is able to rapidly reverse the symptoms of depression [17]. In recent animal studies, ketamine has been found to rapidly increase synapses in the prefrontal cortex (PFC) which sheds light on its effects on neuroplasticity and synaptogenesis [18]. A relatively new technology called the* optogenetic stimulation* of infralimbic prefrontal cortex (IL-PFC) in mice models has been recently shown to reproduce ketamine's rapid and sustained antidepressant actions [19]. This technology is an experimental method that involves the use of light to make specific neurons fire one at a time so that the precise connection between the two brain regions in real time can be assessed and thus it provides a more accurate correlation between neuronal activity and behavior in animal models. These new findings open new vistas to understand the mechanisms behind psychiatric disorders including depression and inform us that new therapeutic approaches including manipulation of this neural circuitry by pharmacological agents like ketamine or by neurostimulation technologies including transcranial magnetic stimulation (TMS) or optogenetic stimulation can have important treatment implications. It is also possible that ketamine may cause inhibition of the activities of the GABAergic (inhibitory) interneurons thereby* increasing* the overall excitatory postsynaptic transmission [1], which may lead to its antidepressant effects. Postmortem studies of depression patients and rodents have found atrophy in the prefrontal cortex (PFC) and hippocampi. These findings are also seen as effects of chronic stress and ketamine has a potential to reverse this atrophy. Of note, ketamine, although a glutamate antagonist, can actually* increase* glutamate transmission in PFC. Ketamine decreases the spontaneous activity of GABAergic interneurons (these interneurons inhibit glutamatergic transmission) in the PFC and thereby results in disinhibition of glutamate transmission. This effect could be dependent on mTOR [18]. Apart from the involvement of the glutamate system, the latest literature suggests an involvement of nicotinic acetylcholine receptors (NAR, specifically its alpha-7 subunit) in TRD [20]. Ketamine (R-S enantiomer) and its metabolites (most importantly the hydroxynorketamine, HNK), in subanesthetic dosages, in both rodents and humans, have been shown to inhibit the NAR, mainly via noncompetitive antagonism of the NAR. They inhibit the acetylcholine evoked currents in the alpha-7 NARs and thus block the signal transduction through mammalian NAR channels by interacting with both their open and closed states.

## 4. Efficacy of Ketamine in TRD: Clinical Data

### 4.1. Single Infusion of Ketamine in TRD

Ketamine acts rapidly and can be administered even orally or intranasally along with its known intravenous and intramuscular routes as an anesthetic agent. However, its oral bioavailability is quite low to be able to exert therapeutic effects. The first double blind placebo controlled trial done by Berman et al. (2000) demonstrated rapid antidepressant effects of single dose of ketamine in seventeen patients with TRD that lasted for 1 week, assessing antidepressant efficacy of single subanesthetic dose of ketamine (0.5 mg/kg administered over 40 minutes) [21]. Ketamine is used in much higher dose range if it is used for anesthesia purposes, that is, 6.5 to 13 mg/kg if used IM and 1 to 4.5 mg/kg if used IV. Since this initial study, this effect of ketamine has been proven in five more recent studies with good methodology that involved patient with unipolar TRD [4, 22–24] and also in patients with bipolar depression [25, 26]. This effect is sustained for approximately 1-2 weeks [27, 28]. Efficacy of ketamine in TRD, compared to other antidepressant medications, stands out in terms of not only its rapidity of effects (within 2–4 hours) but also its ability to rapidly reverse suicidality in patients with TRD [25]. More recently, Murrough et al. (2013) have expanded upon the initial work of Berman et al. (2000) by conducting a two-site, parallel arm randomized controlled trial that investigated the effects of ketamine on an ethnically and racially diverse sample that was relatively large (*N* = 73) [21, 24, 29]. Patients were assessed at multiple time periods (24, 48, and 72 hours and 7 days after infusion) after a single intravenous infusion of 0.5 mg/kg ketamine with 0.045 mg/kg body weight midazolam as a control. After the seven-day mark, the last formal assessment time point, those who responded were followed at every two weeks until relapse. A significant decrease in depressive scores was observed at 24 hours after infusion in the ketamine group compared to control (−7.95 points, 95% CI); responses were maintained beyond the 24-hour mark but were no longer statistically significant at 7 days after infusion. In this study that examined effects of a single ketamine infusion, Murrough et al. (2013) found approximately 60% response rate versus 21% in midazolam group and approximately 46% response rate after 7 days of the infusion versus 18% response in control group. Additionally, although both groups demonstrated an increase in depressive symptoms as a function of time, the ketamine group maintained lower overall depression severity scores. These findings suggest that single ketamine infusion has almost same rapid but transient antidepressant effect as compared to repeated infusion (as discussed in the next section); however, further research with larger studies is needed to assess the efficacy and potency of dosage of ketamine.

### 4.2. Multiple Infusions of Ketamine in TRD

The major limiting factor of ketamine has been its short lasting effects with single dose (usually a few days to one week). This has led to the first clinical trial that examined efficacy of multiple doses of intravenous ketamine [30]. They administered six infusions over two-week period in nine patients with TRD and found mean relapse time of 19 days with one patient having a response for three months which was the longest. This regimen was further studied on larger group of population (24 patients) by Murrough et al. (2013) in a two-site randomized controlled trial; authors not only determined that a series of six ketamine infusions, administered three times weekly over twelve days, demonstrated a high overall response rate (70.8%) but also determined that the median time to relapse among responders was 18 days after the last infusion [24, 29]. Cusin and Dougherty (2012) published two case reports suggesting the potential long term use of intramuscular (IM) ketamine in treatment resistant bipolar depression [9]. They formulated that oral ketamine has poor bioavailability (17–20%) as compared to IM (93%). Interestingly, oral ketamine in the study failed to produce effective antidepressant effects, whereas intramuscular (IM) ketamine was well tolerated with minimal side effects in form of anxiety, headaches, and irritability but significantly reduced the depressive symptoms for months. Their study used 50 mg IM ketamine every 3-4 days for 5-6 months and patients maintained their remissions. These researchers suggested conducting further larger study to confirm the dose and efficacy of IM ketamine as a maintenance treatment of TRD to shed more light on these important aspects [9].

It is important to note that the most studies involving single dosage do not demonstrate a treatment response beyond seven days after single ketamine infusion compared to studies with repeated infusions which show a longer lasting effect, thus extending the time to relapse. There have been some concerns that ketamine (particularly at doses higher than the subanesthetic doses) when given in multiple doses over longer duration of time may cause abuse liability and also may cause some memory impairments that could continue even after ketamine has been stopped. In order to assess safety and efficacy of ketamine in TRD, Diamond et al. (2014) conducted a small, descriptive, open label study (28 patients) and found that ketamine infusions in patients, up to six doses at a time in one course of treatment, are overall safe and well tolerated with very minimal side effects such as anxiety, vomiting, and vasovagal response [31]. In this study, 29% of patients achieved response rate which is exactly the same as that by Aan het Rot et al.'s (2010) review of five RCTs with single ketamine infusion [32]. This study further validates the earlier studies on safety and efficacy of ketamine in TRD and these authors suggested that at least two infusions of ketamine are required to sustain its beneficial antidepressant effects and recommended further research to confirm this finding. Additionally, some of the literature suggests that ketamine may have utility as an anesthetic for electroconvulsive therapy (ECT), such that patients receiving the drug may experience greater improvement in depressive symptoms when compared to use of standard anesthetics [33, 34]. These researchers found that patients receiving a 0.5 mg/kg dose of ketamine experienced a small but significant increase in treatment efficacy after one week that did not extend to additional ECT sessions [33]. By increasing the dosage of ketamine to 1-2 mg/kg body weight, they found that antidepressant effects were significant for the first two sessions of ECT and that there was a more rapid antidepressant response to ECT when compared to the control anesthetic (2-3 mg/kg body weight thiopental) [34]. Additionally, seizure duration in the ketamine group was observed as longer in duration, thereby increasing the exposure to the therapeutic effects of ECT. Yet another therapeutic benefit of ketamine could be effect on ECT-induced retrograde amnesia [35]. According to authors, available human and animal evidences support that ketamine, through its effects on NMDA receptor, could effectively prevent ECT-induced persistent retrograde amnesia. Taken together, the research suggests that ketamine has exciting potential as a rapidly effective antidepressant that is useful both as monotherapy and as an augmenting agent in conjunction with ECT. Further research to establish more data on clinical usefulness and more replicability of these findings are warranted.

### 4.3. Utility of Ketamine in Depressed Patients with Suicidality

Although research on this topic is still evolving, one of the most important clinical implications ketamine could have is for the treatment of suicidality in patients with TRD which is indeed one of the most severe symptoms of MDD. The patients suffering from MDD are more likely to have suicidal thoughts as well as suicide attempts. This risk increases significantly in patients diagnosed with TRD. One randomized controlled trial that used ketamine infusion in 33 such patients found rapid reversal of suicidality in them [25]. In this trial, not only did suicidal ideation improve on all measures within 40 minutes, but moderate effects still remained significant at 230 minutes after infusion. Additionally, among patients who scored high on the Scale for Suicidal Ideation (SSI) at baseline (SSI > 3), 90% of them dropped below 4 on the SSI within the 40-minute interval with 60% reaching SSI scores of zero within the first day. These results, taken together with the significant decreases of suicidal ideation on multiple forms of assessment, strongly suggest the importance of ketamine in treating suicidal patients. Other researchers concur with these findings. In a study (*n* = 26) involving both single and multiple infusions of subanesthetic dose of intravenous ketamine in TRD patients, suicidal ideation was decreased 24 hours after infusion after single dose and at 12 days with multiple infusions [36]. These studies that involved administration of ketamine in multiple doses suggest both immediate and long term effects on suicidal ideations. This is a very important finding in treatment of TRD, where suicidality is a real challenge. Indeed, because the decision-making window for suicide attempts is often ten minutes or less, a fast-acting, effective form of treatment is paramount to addressing the needs of these patients and pertinent studies on effects of ketamine in this specific aspect [25, 36] are really encouraging.

More recently, researchers have indicated that the mystical effects of ketamine might have implications for the existential dimensions of personhood, such as sense of self and purpose; the existential vulnerabilities of depressive symptoms such as hopelessness, suicidality, and guilt then might also be ameliorated by the same mechanism. Thus, there may be a psychological component of the ketamine treatment mechanism [37]. Therefore, the antidepressant properties of ketamine are particularly exciting not only because it has generated an avenue for developing novel treatments for resistant depression but also because of its rapidity of action which can be effectively used in suicidal patients. Some authors have even suggested a possibility of ketamine to be a prototype medication for the class of drugs that rapidly act on TRD [38].

### 4.4. Side Effects of Ketamine Can Be Predictors of Treatment Response

Similar to any pharmacological interventions, use of ketamine also may not be free of any unwarranted effects. Indeed, some of the literature suggests that ketamine can cause several possible adverse effects such as dissociative, psychotic, or psychotomimetic effects [39]. Additionally, some research noted hypotension or hypertension, bradycardia and tachycardia throughout infusion, blurred vision, drowsiness, and feeling unreal [29, 30]. However, these symptoms were transient in nature. Authors addressed the effects of ketamine in nine patients with TRD in a study involving ketamine infusion over twelve-day time span [40]. Patients in this study were administered a battery of multiple measurements addressing psychotic symptoms. No patient verbally reported distressing psychotic effects during the entire twelve-day period, with most of the self-report data indicating that the effects of ketamine were “mildly bothersome.” Overall, the results of this study are consistent with the literature suggesting transient, mildly dissociative effects that are rather acute, temporary, and nondistressing after administration of ketamine in these patients [30].

It is clear that the treatment of depression with ketamine requires careful monitoring of vital signs, at least for the initial infusion. Thus, some of the researches recommend hospitalization for TRD patients receiving ketamine treatment [29, 30], so that vital signs and dissociative symptoms can be closely monitored. While some studies indicate that ketamine treatment is an inpatient only therapy, recent preliminary studies demonstrate the success of ketamine for treating TRD patients in the outpatient setting. According to a study, treatment effects of ketamine in outpatient settings were similar to those demonstrated by inpatient treatment protocols. We have successfully used such an outpatient protocol in 15 patients with chronic and refractory PTSD and comorbid TRD [41]. Overall, these findings have exciting implications for the administration of ketamine and treatment of TRD [42].

There have been some indications that some side effects of ketamine may serve as a biomarker of the treatment response. Data from 108 depressed patients with MDD or bipolar disorder who received single dose of IV ketamine demonstrated that behavioral slowness and dissociative side effects, but not psychotomimetic or sympathomimetic effects, correlated with change in depression scores on the day of infusion and seven days after infusion. Based on these correlations, authors suggested that dissociative side effects could be clinical biomarker to predict ketamine's antidepressant efficacy [43]. While results of these studies that examine its therapeutic effects in TRD are encouraging, many patients experience transient dissociation and psychotomimetic side effects and hemodynamic changes (e.g., increases in blood pressure) that limit its clinical use [44]. Other noncompetitive and more specific NMDA receptor antagonists that are currently being used experimentally have antidepressant efficacy in major depression and are relatively devoid of psychotomimetic and dissociative side effects [4, 28, 45]. The effects of these other antagonists, however, are not as robust as ketamine, which may reflect their decreased binding/affinity for the NMDA receptor complex [30]. Thus, it is unclear at this time if the psychotomimetic sequelae, dissociative experiences, and/or hyperdynamic vital sign changes associated with a subanesthetic dose of ketamine are necessary to achieve antidepressant effects.

Another interesting finding is effect of ketamine on cognition which may serve as another clinical biomarker of response to treatment with ketamine. Citing literature demonstrating the neurocognitive effects of ketamine on healthy volunteers, authors addressed the possibility of short term cognitive disruption during the administration of ketamine for TRD [24]. A study evaluated this effect in a sample of 25 patients [46]. A comprehensive battery of assessments was administered at baseline using various scales measuring executive functioning. At 40 minutes after infusion, which is consistent with the established time point associated with peak potential of psychoactive effects, these scales were administered again to assess the effects on cognition. Ketamine was associated with minimal cognitive effects at 40 minutes. Interestingly, lower neurocognitive scores on processing speed subscales at baseline were associated with greater antidepressant response, suggesting a possible method of identifying patients who may respond to ketamine. In this study, 100% of the subjects classified as treatment responders demonstrated no change on neurocognitive assessment at 40th minute time after infusion, while only 50% of the subgroup demonstrating neurocognitive change demonstrated a significant antidepressant response to ketamine (Fischer's exact test two-sided *p* = 0.027). Taken together, these findings suggest the possibility of characterizing a patient profile of successful treatment responders; specifically, those patients who are low performers on processing speed scales may respond better to ketamine treatment for TRD [46]. These also indicate that specific neurobiological mechanisms might exist among treatment responders and the fact that negative cognitive effects were associated with nonresponse to ketamine indicates that side effects might relate to treatment response. Indeed, the recent literature suggests that both dissociative and psychoactive side effects of ketamine mediate treatment response.

The data on ketamine's side effects and their relationship with clinical effects of ketamine as mentioned above seem quite interesting. A study published in 2013 by Sos et al. indicated correlation between ketamine's effects on depression and its psychomimetic effects and also suggested that the NMDA receptors could be mediating such correlation [47]. One can see that both the clinical effects and side effects of ketamine may be governed by common mechanisms. It is also possible that these biological mechanisms of action of ketamine might explain the side effects. As previously mentioned, it has been suggested that ketamine might inhibit GABAergic cortical interneurons, further depolarizing pyramidal neurons and allowing for greater long term potentiation of* downstream* glutamate release, thereby enhancing dissociative effects [43]. Dopamine transmission and the neural circuits of dorsolateral prefrontal cortex and striatum might also be modulated by ketamine and may explain the neurocognitive effects of treatment [46]. Both mechanisms have been implicated in the underlying causes of depression. Therefore, research investigating the biological mechanisms behind the side effects of ketamine not only has the potential to elucidate the therapeutic mechanism of action of ketamine but could also provide additional insight into the neurobiology of depressive disorders in general.

There have been some concerns raised about ketamine's abuse potential especially resulting from the recreational use/abuse of ketamine (i.e., Special K) [48]. However, some researchers do not agree with this and report data to the contrary [42]. This includes a 1-year follow-up study that involved use of single infusion of subanesthetic dose (0.5 mg/kg body weight) of ketamine in 15 patients with refractory PTSD comorbid with TRD. Of note, patients in this study did not have any significant side effects of ketamine [41]. Some studies indicate use of ketamine in TRD to be relatively safe [31]. However, this topic needs further investigations through future research.

To sum up, some concerns at this about ketamine are possibility of side effects, abuse potential especially with more frequent administrations, and the paucity of efficacy research on its long term utility in treatment of TRD. Additionally, the maintenance phase data on its efficacy is sparse. The literature to date reflects mostly on the role of ketamine in acute phase treatment of depression and the long term effects of ketamine use for TRD have not been addressed that much [30]. Although several studies document ketamine's ability to produce sustained antidepressant states for several days after infusion [29, 40, 46], none of the effects have lasted longer than 2 weeks. Thus, despite the enthusiasm about the quite rapid and unexpected efficacy of ketamine, its widespread use as a fast-acting antidepressant in routine clinical settings is curtailed by some of these limitations. However, from early phase data, it does appear that the potential benefits of ketamine may outweigh the potential risks, but this has to be confirmed in long term studies that need to include investigational use of ketamine in maintenance phase of TRD as well. Taken together, the potential for ketamine's utility in acute phase treatment of TRD is robust and side effects for treatment have been mild and transient. Therefore, despite the concerns mentioned earlier, the potential benefits of ketamine as an acute phase treatment option for TRD outweigh the involved risks and clearly warrant further investigation to address the lacunae in the existing knowledge base. One potential way to go could be to use ketamine either alone or in combination with conventional antidepressants or ECT for rapid induction of remission of the acute symptoms of TRD and then use other treatments like conventional antidepressants or cognitive behavioral therapy (CBT) or interpersonal therapy (IPT) or mindfulness based cognitive therapy (MBCT) to maintain the remission as well as to prevent relapse. Another potentially therapeutic use could be to combine ketamine with lithium for inducing quicker remission of TRD symptoms: as mentioned earlier, ketamine and lithium have synergistic effect with respect to their influence on the glycogen synthetase kinase-III enzyme which has been implicated in depression. Thus the ability of ketamine to produce an ultra-fast-acting antidepressant response in depressed patients not only provides a unique opportunity for treatment of resistant depression but also opens the door to further understand the cellular mechanisms that mediate these clinically relevant behavioral effects.

## 5. Repetitive Transcranial Magnetic Stimulation (rTMS) in TRD

Repetitive transcranial magnetic stimulation (rTMS) is a noninvasive brain stimulation technique based on generation of strong magnetic field to stimulate the cerebral cortex without invasion. Usually rTMS is used in treatment of acute TRD and has been approved by FDA in 2009 for this indication. Hypoactivity in left frontal lobe and hyperactivity of right frontal lobe have been postulated as one of the underlying pathologies for depression [9]. Therefore, suppressive rTMS to the right prefrontal cortex and high frequency rTMS to the left prefrontal cortex are believed to be probable primary mechanisms and rationale for the use of TMS in depression. TMS is a US FDA-approved treatment for major depressive disorder (MDD) in patients who have not responded to 1 adequate antidepressant trial in the current episode. TMS is considered to be not only effective but also relatively very safe compared to other forms of treatments [49].

Earlier it was believed that effects of rTMS are short lasting. However, rTMS using H-Coil could have potential effect up to eighteen weeks according to one study [50]. Recent reports suggest that TMS might be beneficial up to 12 months duration. A recent multisite observational study involving a total of 42 sites in the US was conducted in 2014 by Dunner et al. [51]. All sites used the same TMS machine and the same standard treatment parameters. These parameters included 120% motor threshold, pulse frequency of 10 pulses per second with each cycle of 4 seconds with active stimulation, and 26 seconds with no stimulation. This was repeated for a total of 75 cycles resulting in total 3000 pulses per session. Each session lasted for 37.5 minutes. This study suggests and supports the ongoing norms of effectiveness of TMS as an acute treatment for TRD and particularly this study also establishes the effectiveness of TMS as maintenance treatment of TRD up to 12 months of duration particularly in patients who have received initial acute response with TMS. According to this study, lower chances of relapse are reported, but a major limitation of this study is the study design which was observational [51].

A pilot study [52] demonstrated clinical effectiveness of TMS in TRD. A randomized controlled trial (*n* = 179) compared bilateral versus right frontal rTMS in TRD patient. This study demonstrated encouraging results in terms of improvements assessed with Hamilton Depression Score but did not find any difference in beneficial outcome based on site of TMS. In other words, bilateral TMS was not found to be more effective compared to right-sided TMS. A meta-analysis of 18 TRD studies published between 1980 and 2013 to evaluate effect of TMS showed that, in a group of MDD patients with two or more antidepressant failures, rTMS is an effective option [53]. A systematic review of TRD in adolescents found TMS to be effective and well tolerated [54]. Yet another area of research includes bilateral versus unilateral rTMS in TRD. A meta-analysis assessed and compared 10 RCTs and it was found that bilateral rTMS showed increase in response and remission compared to unilateral rTMS; however, the increase was found to be statistically not significant. Interestingly, compared to sham rTMS, bilateral rTMS was found to be more effective but not unilateral rTMS. This area still remains in need for further research [55]. Various additional advantages of rTMS are also reported in the literature. One of the meta-analyses suggests use of rTMS to be effective as augmentative approach to TRD [56]. Another meta-analysis suggests potential use of TMS in patients who would be candidates for ECT [57]. A recent review article reports possible cognitive enhancement with rTMS [58].

### 5.1. Safety Profile and Limitations of rTMS

Adverse reactions of rTMS have been studied very well in last decade among various studies. Overall, rTMS procedure is well tolerated with no cognitive impairment and no induction of seizures even with high frequency TMS. It has been reported that low frequency rTMS may be protective against seizures. No serious adverse events are reported. Mild side effects such as headache and dizziness are well known. Overall, TMS has a favorable side effect profile compared to ECT and medications. However, rTMS cannot be used in certain situations and is in fact contraindicated, and, in certain conditions, the usage is recommended with caution [59]. In line with US Food and Drug Administration's Center for Radiological Health guidance on rTMS, some of the contraindications for TMS include (1) presence of metallic objects in or near head (within 30 cm of the coil), for example, cochlear implants, implanted electrodes/stimulators, aneurysm clips or coils, stents, bullet fragments, jewelry, and hair barrettes, with standard amalgam dental fillings, single postdental implants, and dental bridge work not being the contradictions for TMS use and (2) patients who have active or inactive implants (including device leads), including deep brain stimulators, cochlear implants, and vagus nerve stimulators.

Caution is required in patients with (1) pacemakers and implantable cardioverter defibrillators (ICDs) or patients using wearable cardioverter defibrillators (WCDs) even if the device is removed due to the potentially unstable cardiac condition of such patients, (2) staples and implanted insulin pumps anywhere in body, (3) history (or family history) of seizure or epilepsy, history of stroke, head injury, severe headaches, or unexplained seizures, (4) presence of other neurological diseases that may be associated with an altered seizure threshold (such as CVA, cerebral aneurysm, dementia, increased intracranial pressure, head trauma, or movement disorder), (5) concurrent medication use such as tricyclic antidepressants, neuroleptic medications, or other drugs that are known to lower the seizure threshold, (6) secondary conditions that may significantly alter electrolyte balance or lower seizure threshold, and (7) no quantifiable motor threshold such that TMS dosage cannot be accurately determined.

In summary, even though there is fairly good amount of literature supporting role of rTMS in MDD and TRD, its use in TRD needs more investigation [60, 61]. The efficacy of rTMS in TRD has been studied with the combination of antidepressant drugs and even the positive results have been reported on studies with small sample size and variable inclusion criteria. Thus, role of TMS in the treatment of TRD is still under investigation based on published data [9]. Despite milder side effect profile compared to other modalities for depression, few important limitations are as follows. (1) rTMS may not be that convenient to patients with TRD. Patients require daily stimulations for 25–30 sessions requiring outpatient office visits. (2) Its comparative efficacy is around 45–55% at the most. (3) Although it is being increasingly reimbursed by the health insurance companies, there are affordability issues. (4) Data on its long term efficacy as well as on maintenance phase of depression are still sparse. For these reasons, treatment modality that is highly effective, offers long term help, and can be administered in home settings is important. Yoga and mindfulness based interventions could fill these gaps to some extent and can be used as adjuvant modalities.

## 6. Yoga and Mindfulness Based Interventions in TRD

Despite their efficacy especially in the acute phase treatment of depression, in light of the existing limitations of rTMS and psychopharmacological approaches including ketamine, the investigation of alternative forms of treatment needs serious attention. Yoga and mindfulness are probably among the most ancient forms of mind-body medicine interventions that have shed light on the intricate and complex dynamic interplay between the body and mind with clear outlines and methods about how one can achieve physical, mental, and spiritual well-being. Yoga and meditation, as used in the West, fall under the broad rubric of complementary and alternative medicine (CAM). As elaborated in the meditative philosophies passed down since a few thousand years back, when the individual's (Sanskrit:* jiva*) actions are governed by the meditative insights (which results in* wisdom*) rather than just as reactionary responses to the underlying impulses, these* wise actions* do not bring about* suffering* (Sanskrit:* dukkha*, which literally means* sadness* as well) as described by Buddhaghosha [62]. Yoga and mindfulness interventions, especially when they are used in a symptom targeted way, have relatively low cost, are not known to have any serious side effects, have clear biological rationale (especially with the recent insights from neuroimaging as well as from RCTs) in altering the stress diathesis model that could influence the onset as well as relapse of depressive episodes, and most importantly have utility as self-management techniques which empowers the person as a whole rather than (rather narrowly) affecting just the symptoms of depressive disorder [12, 13, 63–65]. These are strong rationale not only to study these interventions further in TRD, because of fairly good emerging evidence that supports their utility, but also to clarify further on the mechanisms of their actions and also to estimate a dose-response relationship, if any, between the various MBCT interventions and TRD. Utility of Yoga and meditation in depression offers some promise in the various research studies [66]. Like the cognitive behavioral therapy (CBT), mindfulness based cognitive therapy (MBCT) functions on the theory that when individuals who have historically had depression become distressed they return to automatic cognitive processes that can trigger a depressive episode. The goal of MBCT is to interrupt these automatic processes and teach the participants to focus less on reacting to incoming stimuli and instead focus more on accepting and observing them without judgment. This mindfulness practice allows the participant to notice when automatic processes are occurring and to alter their reaction to be more of a reflection [67]. Over the last decade, numerous studies have examined the efficacy of mindfulness based cognitive therapy (MBCT) in depression. Many of the studies have demonstrated the efficacy of MBCT in prevention of relapses [68, 69], and more recently there have been attempts to study its effects on acute phase depression [68, 70, 71]. Another recent meta-analysis examined use of MBCT for depression in six randomized controlled trials with a total of 593 participants [72]. This meta-analysis showed that MBCT significantly reduced the risk of relapse/recurrence with a risk ratio of 0.66 for MBCT compared to treatment as usual or placebo controls, corresponding to a relative risk reduction of 34%. Results of this meta-analysis indicate that MBCT is an effective intervention for relapse prevention in patients with recurrent MDD in remission, at least in case of three or more previous MDD episodes.

### 6.1. MBCT versus Yoga and Mindfulness Based Cognitive Therapy (Y-MBCT, Pradhan, 2014, 2015)

It is important to understand that* Yoga*,* meditation*, and* mindfulness* are conceptually three overarching circles in the broad scheme of Yoga. Yoga* in its entirety*, as it was proposed originally (in ancient India), consists of eight limbs which includes meditation as its two crucial steps, that is, 6th step (concentrative type meditation, Pali:* samatha*, Sanskrit:* dharana*) and 7th step (mindfulness type meditation; Pali:* satipatthana*,* vipassana,* Sanskrit:* dhyan*) [62, 65, 73]. Meditation is essentially an ongoing cognitive-emotive-reappraisal process that takes place within the individual in order to obtain insight and* directly* experience the various mind-body phenomena first hand, without trying to alter (distort) them. Technically, meditation* involves learning to shift and focus one's attention at will onto an object of choice*, such as bodily feelings or an emotional experience, while disengaging oneself from their elaborative processing by the mind.

When one examines the patterns of clinical use of Yoga and meditation in the Western world, interestingly one comes across their rather dichotomous use in contrast to how they were purported to be used in the ancient spiritual traditions. In these modified forms, Yoga is used mostly as a physical exercise, whereas meditation is confined to its use as a mental technique and thus when used in piecemeal the scope and utility become rather narrow and the integration between body and mind is far from being complete. Mindfulness interventions, as they are clinically used in the West, are heterogeneous and usually are secular amalgamations of the various Buddhist practices, that is, Zen, Tibetan Buddhism, and Burmese spiritual practices. Compared to the dichotomous use of Yoga as mentioned before, the Yoga and mindfulness based cognitive therapy (Y-MBCT) models use Yoga* in its entirety* rather than its piecemeal use as just a physical exercise or a breathing technique or only mediation and so forth. Thus the components in the Y-MBCT include all eight steps of Yoga involving body and mind. This broader and integrated application not only increases the scope of these interventions but also increases their efficacy. These models incorporate* the balanced* life style, posture (asana), Yogic procedures (kriya), and, of course, meditation in their standardized forms. The Y-MBCT models combine Yogic* philosophies*,* techniques*,* and practice* into pragmatic and user-friendly formats. The cornerstones of the Y-MBCT models are the Staged Meditation Protocols (SMPs), the balanced and meditative lifestyle (Buddha's* Middle Way*, which advocates for lifestyle and life views of* moderation* and* compassion* rather than extremes and self-mortification and criticality), meditative breathing (calming and energizing types), and symptom specific Yogic procedures (Sanskrit:* kriyas*). Psychotherapeutic use of the Y-MBCT models is based on* two major themes in Yoga*: (i)* Yoga as a profound psychosomatic science* and (ii)* meditation as a science of attention*. The Y-MBCT models are translational, targeted, and therapeutic, that is, rooted in the ancient wisdom of the meditative traditions but use methodology of the evidence based medicine. These models are based on the fundamental philosophies about human experiences and outline that the pathological experiences in the subjects are deranged and dysfunctional and are exaggerations of the normal human experiences. Thus the disorder specific models in Y-MBCT are extensions and modifications of the normal experiences. Conceptually, these treatment models are based on universality of* both normal and pathological* human experiences, are less stigmatizing, and serve an important purpose of establishing empathic relationship with the patient and do not alienate the patient from the therapist. The Y-MBCT model is more experiential and this therapist needs to be a practitioner; that is, he/she practices the wellness model first and also in the session the therapist practices with patient. The Y-MBCT interventions are more inclusive and holistic and targeted towards the whole person and the associated clusters of symptoms.

### 6.2. Brief Description of the Depression Specific Y-MBCT (*DepS Y-MBCT*) Model and Its Psychosomatic Adaptations

**Figure 1:**
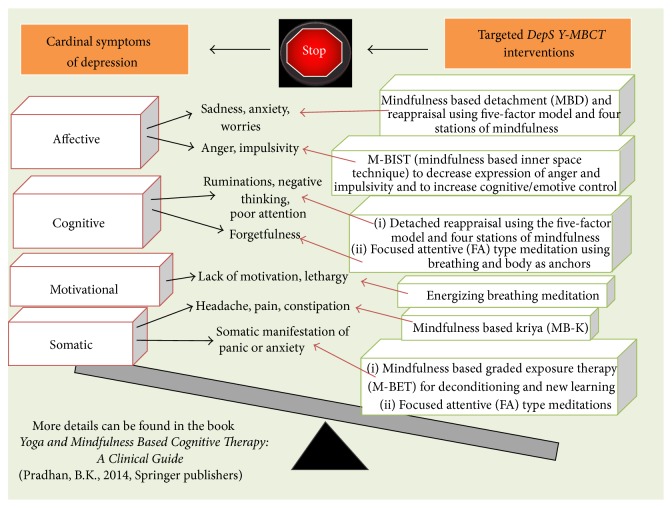
Depression specific Yoga and mindfulness based cognitive therapy (*DepS Y-MBCT*) model.

*DepS Y-MBCT* is a targeted approach and conceptually and methodologically, like the DBT or ACT or MBCT, it falls under the broader category of the* third wave cognitive therapy* [74]. The five-factor model [65] describes the human experience in terms of five things in combination, that is, one's thoughts, feelings, the attending sensations or perceptions, the will or urges or impulses that are aroused by these thoughts or feelings, and finally associated memories that serve as a cementing substance, and provides context to the other four elements of the five-factor model. The quantitative* five-factor inventory* developed by the first author based on this five-factor model provides data on the depression experience of the patient which are targeted by the symptom specific mindfulness interventions using the tools of brief cognitive behavioral therapy (CBT) in an experiential manner as depicted in Figure 1 below. The main tools involved in the* DepS Y-MBCT* are

- Calming and energizing breathing types to be practiced in sitting/lying down posture.
- Staged Meditation Protocols (SMPs, levels 1, 2, and 3): this involves both the focused attention (FA) type meditation that uses breath and body as the anchors to focus and induce detachment from the symptoms and the open monitoring (OM) type meditation empowered by the philosophy of the five-factor model and breathing meditations that maintain the detachment from the cardinal symptoms of depression and enhance detached monitoring and reappraisal of symptoms and thus promote new learning.
- The* kriyas* ([Sanskrit] the Yogic procedures) and their symptom specific psychosomatic adaptations for use in the somatic symptoms of depression.

Daily home practice (routinely and as needed) and flexibly adopting a balanced life style (using the philosophy of Middle Way) provide the matrix for generalization of these interventions to the subject's daily life.* DepS Y-MBCT* training and practice are neither religious nor physically demanding at all. Rather it is a mental training using Standardized Meditation Protocols (SMPs) and so just a comfortable posture and establishing a targeted and regular home practice routine are all that is needed. All interventions in the* DepS Y-MBCT* are personalized by the mindfulness profile of the patient which is assessed by a quantitative scale, Assessment Scale for Mindfulness Interventions (ASMI) [65]. This is an 18-item scale which identifies 7 core dimensions of mindfulness and its scores range from 0 to 90: the higher the scores are, the more the level of mindfulness in the subject is. Thus ASMI scale quantitatively assesses the level of mindfulness in the subject (before and after intervention) and helps to individualize the* DepS Y-MBCT* interventions for each subject while keeping the overall format of therapy intact.

### 6.3. Preliminary Data on Efficacy of* DepS Y-MBCT* in TRD

*DepS Y-MBCT*, as a psychotherapy, has been studied in a population with diverse cultural or ethnic background suffering from nonpsychotic depression (both unipolar depression and bipolar depression) with or without comorbid anxiety disorders [13]. In this pilot study involving open label trial, prospective, design, and sequential recruitment of the study subjects, we hypothesized that self-exploratory and more experiential psychotherapy after initial training of them by an expert therapist when done in a specific and standardized way to target the cardinal symptoms of depression (as done in the* DepS Y-MBCT* model) may augment antidepressant medication(s) that were not effective in the subjects prior to study entry. The sample in this study consisted of a population with diverse cultural or ethnic backgrounds of 34 patients, with ages ranging from 14 to 64 years from both genders. Out of 34 patients with TRD, no patient refused participation in this study but two patients dropped out before completing initial three training sessions of* DepS Y-MBCT* and did not return our phone calls asking to let us know about the reasons for the dropout. This open trial was done from September 2012 to December 2014 at the Outpatient Psychiatry Clinic of the Cooper University Hospital, Camden, NJ. Diagnosis of TRD and other psychiatric comorbidities was done by a 90-minute comprehensive initial psychiatric evaluation by the first author (BP) who did review of medical records, used clinical interview using DSM-IV criteria and the definition of TRD (failure of an adequate trial of at least 2 antidepressant medications, as mentioned in the Introduction), and used rating scales, that is, Ham-D (17 items) and ASMI. As defined by these parameters, all patients had symptoms of severe major depression without current psychotic features and 11 out of 32 subjects had suicidal ideas without any plan. As discussed earlier,* DepS Y-MBCT* interventions are mostly mental training and therefore not physically demanding. Therefore, even though subjects with TRD are considered very sick to engage in therapy in general, they did not have much difficulty in engaging in these interventions. Additionally, mindfulness profile of the subjects as measured by the ASMI scale helped personalize these interventions and thus enhanced their feasibility in this cohort of subjects. The mean duration of depression in these subjects was 11.2 (SD 4.3) months. Their mean depression severity scores (as assessed by the Ham-D, 17-item scale) at study entry were 24.3 (SD 6.7) which falls in the “severe” range [75]. The level of mindfulness of subjects (as measured by their mean ASMI scores) at study entry was 46.3 (SD 5.3). Patients with current psychotic features or suicidal plan or active manic symptoms or drug dependence were excluded from this study. In 11 out of the 32 subjects, because of request from patient/family, antidepressant medications were continued and their dosages were kept unchanged throughout this trial and no new antidepressant medications were added. Of note, before their enrolment into the* DepS-Y-MBCT* interventions, these subjects were experiencing persistent symptoms of depression despite taking these medications in adequate dosage and duration. All* DepS-Y-MBCT* sessions were individual sessions (45 minutes each, once every 1-2 weeks, carried out in the first author's outpatient office) along with twice daily* routine home practice* (15–20 minutes each) of standardized Staged Meditation Protocols (SMPs) to master the five-factor model and detached reappraisal of the symptoms and the accompanying distress. Additionally, subjects practiced the* as needed home practice* (5 minutes each, usually 3-4 times daily) to handle the outburst of emotional and behavioral symptoms as they arise during the subjects' daily life situations. Stable remission was defined as reduction of Ham-D: 17-item scores to ≤7 and maintenance of scores at this level for at least 8 weeks. The number of therapist assisted* DepS Y-MBCT* sessions needed for remission was 9.6 (SD 3.5). Out of the 32 subjects who participated in this study, 27 subjects have completed the whole duration of treatment and have remitted from acute symptoms of depression as defined by their clinical improvements as well as reduction of their Ham-D scores to ≤7. The level of mindfulness of these study subjects (as measured by their mean scores on ASMI scale) at remission increased to 69.4 (SD 8.5). Of note, the mean ASMI scores at study entry were 46.3 (SD 5.3). Out of the 11 subjects that chose to continue their antidepressant medications, one subject needed administration of seven rTMS sessions (to left PFC) in addition to* DepS Y-MBCT* interventions for induction of remission. None of these subjects needed psychiatric hospitalization throughout course of treatment. At this time, 11 patients have been discharged to the care of their primary care physicians or therapists with sustained remission of symptoms for more than 4-month duration. There was high acceptability, feasibility, and patient satisfaction in the patients who continued the* DepS Y-MBCT*. Additionally, mindfulness interventions done via* DepS Y-MBCT* could increase the level of mindfulness in the subjects as evidenced by increase in the postinterventions scores of ASMI as compared to the baseline and also in their core dimensions of mindfulness that include their level of attention, feeling of compassion towards self and others, and ability to stay in present moment and nonjudgmental attitude. We believe these core changes might have contributed to their clinical and functional improvements as well.

### 6.4. Limitations of This Pilot Study

The main limitations of this study are its small sample size, open trial, lack of randomization, and the fact that 11 patients in this sample were continued on their antidepressant medications which were previously ineffective. However, there remains the possibility that* DepS Y-MBCT* could have sensitized these patients to previously ineffective antidepressants which might have played a role in their positive treatment response. This is only a pilot study and we intend to use a randomized controlled design in future. We are aware of the need for replication of the preliminary data on efficacy of the* DepS Y-MBCT* model in future studies. Although these efficacy data are promising, use of psychotherapy as a sole modality of treatment in patients with TRD is not without risk or harm. It can be argued that offering only psychotherapy to patients with TRD may let them think that their symptoms were not taken seriously and also previous negative experience with psychotherapy may prevent a patient from engaging in an otherwise proven psychotherapy like CBT. In our study, many patients preferred to have no medications which could have originated from their frustrations from failures of repeated trials of these medications. Additionally, patients in our study expressed high level of satisfaction and their follow-up and remission rates were rather high. So it seems unlikely that the above factors played out in these patients enrolled in our study.

### 6.5. Discussion and Our Suggestion for Adopting a Tiered Approach for Treatment of TRD

Main drawback in the treatment of treatment resistant depression is the limited options, scarcity of knowledge about optimal treatment regimen, and inconsistency in the definitions of TRD. These pose significant challenges in operationalization and management of patients with TRD [5]. There are several risk factors and comorbidities such as CAD, hypothyroidism, type 2 diabetes, chronic pain, sleep disorders, obesity, and medications like interferon and steroids, and anxiety, substance use, personality disorders, adverse life events, and low socioeconomic status are associated with TRD which make it more resistant to the treatment [7]. The existing limitations in treatments for TRD have opened doors to many protocols for new medications and alternative treatment approaches. Multiple therapeutic options with benefits and risks have also led to investigation of therapies that are nonconventional and combine two or more approaches. Both ketamine and TMS provide important immediate and short term benefits in TRD. However, current knowledge is not enough for ketamine to be introduced yet in routine clinical practice [76]. As a treatment modality for depression, rTMS requires 5 times a week treatment sessions for about 25–30 sessions which may pose difficulties for patients suffering from TRD not only in terms of attending so many sessions but also in terms of affordability issues. Compared to this,* DepS Y-MBCT* requires weekly sessions for only initial three to four training sessions followed by 4-5 sessions every two weeks. Once a targeted and symptom specific home practice is established, patients can be transitioned to once monthly sessions. It is also important to note that the various components of* DepS Y-MBCT* (like breathing meditation and focused attention meditation) can be combined with rTMS during a treatment session of rTMS because during administration of rTMS the patients are awake. As noted above in the previous section, we have tried this combination in one patient (she required* DepS Y-MBCT* with seven rTMS sessions).

Another important point is that there has been emergence of literature support that sheds light on putative neural mechanisms that could be involved in actions of rTMS, ketamine, and Yoga and mindfulness based cognitive therapy. Glutamate and GABA in the frontothalamic network have been proposed as two such chemicals. In particular, the increased release of GABA has been demonstrated in the Magnetic Resonance Spectroscopy (MRS) studies after mindfulness based interventions [77, 78]. Based on the literature evidence, as we have presented in this paper, the emerging albeit preliminary neurobiological evidence, the preliminary data on efficacy of* DepS Y-MBCT*, we suggest a “tiered approach of treatment for TRD” in which these treatment modalities can be combined. Considering the limitations of existing treatment options including those of ketamine and rTMS when used as the sole modality of treatment, one way of executing this “tiered approach for TRD” could be to use ketamine or rTMS (alone or along with antidepressant medication) for rapid induction remission of acute symptoms of depression and to use* DepS Y-MBCT* for maintaining remission and preventing relapse. Needless to say that our field of psychiatric therapeutics is in dire need of studies with good methodology that can further examine the validity of these hypotheses which will hopefully provide some respite for the plight of patients with such a debilitating condition as TRD. In addition, such studies may help clarify further the underlying mechanisms of depression and its myriads of manifestations.
